# Depth-Resolved Attenuation Mapping of the Vaginal Wall under Prolapse and after Laser Treatment Using Cross-Polarization Optical Coherence Tomography: A Pilot Study

**DOI:** 10.3390/diagnostics13223487

**Published:** 2023-11-20

**Authors:** Ekaterina Gubarkova, Arseniy Potapov, Alexander Moiseev, Elena Kiseleva, Darya Krupinova, Ksenia Shatilova, Maria Karabut, Andrey Khlopkov, Maria Loginova, Stefka Radenska-Lopovok, Grigory Gelikonov, Gennady Grechkanev, Natalia Gladkova, Marina Sirotkina

**Affiliations:** 1 Institute of Experimental Oncology and Biomedical Technologies, Privolzhsky Research Medical University, 603950 Nizhny Novgorod, Russia; 2 Center of Photonics, Lobachevsky State University of Nizhny Novgorod, 603950 Nizhny Novgorod, Russia; 3 Institute of Applied Physics of the Russian Academy of Sciences, 603950 Nizhny Novgorod, Russia; 4 Department of Obstetrics and Gynecology, Privolzhsky Research Medical University, 603950 Nizhny Novgorod, Russia; 5 Nizhny Novgorod Regional Oncologic Hospital, 603126 Nizhny Novgorod, Russia; 6 “MeLSyTech” Ltd., 603901 Nizhny Novgorod, Russia; 7 Institute of Clinical Morphology and Digital Pathology, I.M. Sechenov First Moscow State Medical University, 119991 Moscow, Russia

**Keywords:** pelvic organ prolapse (POP), vaginal wall prolapse, lamina propria, connective tissue, collagen bundles, neodymium (Nd:YAG) laser treatment, cross-polarization optical coherence tomography, attenuation coefficient, lymphatic vessels

## Abstract

Vaginal wall prolapse is the most common type of pelvic organ prolapse and is mainly associated with collagen bundle changes in the lamina propria. Neodymium (Nd:YAG) laser treatment was used as an innovative, minimally invasive and non-ablative procedure for the treatment of early-stage vaginal wall prolapse. The purpose of this pilot study was to assess connective tissue changes in the vaginal wall under prolapse without treatment and after Nd:YAG laser treatment using cross-polarization optical coherence tomography (CP OCT) with depth-resolved attenuation mapping. A total of 26 freshly excised samples of vaginal wall from 26 patients with age norm (*n* = 8), stage I–II prolapses without treatment (*n* = 8) and stage I–II prolapse 1–2 months after Nd:YAG laser treatment (*n* = 10) were assessed. As a result, for the first time, depth-resolved attenuation maps of the vaginal wall in the B-scan projection in the co- and cross-polarization channels were constructed. Two parameters within the lamina propria were target calculated: the median value and the percentages of high (≥4 mm^−1^) and low (<4 mm^−1^) attenuation coefficient values. A significant (*p* < 0.0001) decrease in the parameters in the case of vaginal wall prolapse compared to the age norm was identified. After laser treatment, a significant (*p* < 0.0001) increase in the parameters compared to the normal level was also observed. Notably, in the cross-channel, both parameters showed a greater difference between the groups than in the co-channel. Therefore, using the cross-channel achieved more reliable differentiation between the groups. To conclude, attenuation coefficient maps allow visualization and quantification of changes in the condition of the connective tissue of the vaginal wall. In the future, CP OCT could be used for in vivo detection of early-stage vaginal wall prolapse and for monitoring the effectiveness of treatment.

## 1. Introduction

Pelvic organ prolapse (POP) is a condition when organs inside the pelvis drop due to weakening of the supporting muscles and tissues of the vaginal wall. The prevalence of POP increases with age, affects the quality of life of the patients and can lead to social problems [1]. The cause of prolapse is multifactorial but is primarily associated with pregnancy and vaginal delivery, which both lead to direct pelvic floor muscle and connective tissue/collagen injury [2,3,4]. Epidemiological data also point to a genetic predisposition (polymorphisms of collagen-related and extracellular matrix-remodeling genes) and, therefore, the need for early detection of POP and timely treatment [5].

Normal pelvic organ support is provided by the interaction between the levator ani muscles and the connective tissues that attach the uterus and vagina to the pelvic side walls [6]. In the case of dysfunction of the supporting apparatus, the vaginal wall is subjected to pressure from the other pelvic organs, which leads to prolapse of the anterior, posterior or apical segment of the vagina. While pelvic floor muscle insufficiency is usually associated with vaginal birth and injury to the levator ani muscles [6,7], connective tissue insufficiency is explained by changes in the content, structure, biomechanics and catabolism of collagen [8,9]. It is collagen, organized into fibers and bundles, that normally provides tensile strength to tissues. It has previously been demonstrated that vaginal wall thickness decreases in women with prolapse, and this may be due to changes in the collagen bundles [10]. Histological changes in pelvic floor tissue showed that collagen fibrils lost their native well-organized, tightly packed morphology and acquired loose, disorderly and discontinuous structures [11]. However, research into changes in collagen bundles in the tissue of the vaginal wall is limited and controversial [12]. We suggest that studies using new high-resolution optical techniques to assess collagen bundle changes in the vaginal wall would be useful.

Further issues requiring discussion are the treatment options for POP. The vaginal wall is the component of the pelvic support system that is targeted for treatment in the early stages of POP. Traditional treatments include observation, physical therapy for the pelvic floor, vaginal pessaries and surgery, but these approaches often do not provide entirely satisfactory results and can even cause urological problems [13,14,15,16]. Currently, the alternative to such traditional treatments for patients with early-stage POP is provided by various laser technologies (ablative and non-ablative laser exposure), these being highly effective and minimally invasive during treatment [17,18,19,20,21]. However, laser treatment does not provide complete recovery, but it enables strengthening (tissue compaction) of the vaginal wall by remodeling the collagen framework to improve the results of further surgical treatment.

Intravaginal neodymium laser (Nd:YAG) with a wavelength of 1064 nm provides innovative, minimally-invasive non-ablative treatment of early-stage vaginal wall prolapse. The advantage of this approach compared to other laser technologies is that the treatment uses a photothermal effect. Laser radiation is absorbed predominantly by the deoxyhemoglobin and oxyhemoglobin of the erythrocytes in the microvasculature, causing a controlled increase in the temperature of the connective tissue of the vaginal wall and activating overexpression of heat shock proteins [22,23,24]. The result of such thermal exposure is the remodeling of collagen bundles in addition to activation of angiogenesis and collagenogenesis [25], which leads to an increase in the density of the connective tissue and, accordingly, the supporting function of the vaginal wall [26]. In addition, due to the low absorption by the main components of the tissue (water and collagen), the effect is carried out over the entire depth of the mucosa (several millimeters), therefore including the lamina propria, whereas classical ablation lasers create micro-damage in the surface layer to the depth of only a few microns [27].

A number of studies have demonstrated the initial results of the clinical efficacy of Nd:YAG lasers in gynecology [28,29,30]. The advantages of this type of laser are the non-invasive exposure, the absence of complications and the reduction of the rehabilitation period to 1 week from 1 month following ablative laser exposure. At the same time, however, the lack of an objective method for monitoring the efficiency of Nd:YAG laser treatment has so far prevented its wide clinical application. It is necessary to use new high-resolution and non-invasive methods for visualization of the microstructure of the vaginal wall in order to obtain objective assessments of pathological changes in the lamina propria connective tissue before and after laser treatment without the need to take biopsies.

Optical coherence tomography (OCT) is a high-resolution (5–15 μm) optical imaging technique that can be clinically used for noninvasive assessment of the cervical and vaginal mucosa to an image depth of 1.5 mm—approaching the biopsy depth for histological examination and the corresponding depth of neodymium laser exposure. In earlier studies, conventional OCT has been successfully used to quickly identify vaginal epithelial damage [31], for monitoring its treatment with nonoxynol-9 vaginal gel [32] and for analysis of human ovarian tissue [33,34]. The first encouraging results of using OCT in vivo for tracking vaginal tissue changes after treatment with fractional-pixel CO_2_ laser therapy for genitourinary syndrome of post-menopausal women have also been obtained [35,36]. The emerging OCT modalities, such as polarization-sensitive (PS) OCT, OCT-angiography and OCT-elastography, as well as endoscopic and handheld scanning probes, significantly extend the prospects and possibilities of the application of OCT techniques to various types of tissues and organs [37,38,39]. More recently, it has been shown that compression optical coherence elastography (C-OCE) can provide a robust method for the detection of the early stages of vaginal wall prolapse based on significant decreases in tissue stiffness of the vaginal wall and can be used for assessing the increase in stiffness of the wall after laser treatment [30].

PS OCT mode allows qualitative and quantitative assessment of the collagen fibers’ state (optically anisotropic structures) in different types of biological tissue using birefringence properties. Birefringence is a phase retardation change with depth which characterizes orientation and spatial collagen fiber organization [37]. Cross-polarization OCT (CP OCT) is a variant of PS OCT that allows imaging of collagen fibers state not only due to its birefringence but also cross-scattering properties. CP OCT devices have two channels to register the backscattered light: co-channel for waves which retained the initial polarization state and cross-channel for waves with a changed polarization state to the orthogonal one. It has been shown that OCT signal intensity in cross-polarization channels depends on the thickness, orientation and density arrangement of collagen fibers [40]. The attenuation coefficient of the OCT signal is commonly used to quantify the CP OCT data. It helps to improve the assessment of the collagen state, for example, in non-tumorous and tumorous tissues [41], in the identification of an early stage of vulvar lichen sclerosus [42], and in the detection of areas of inflammation vs. norm and necrosis [43]. More recently, it became possible to build depth-resolved attenuation maps in the B-scan projection for the earlier detection of ovarian and fallopian tube malignancy [44]. It has been shown that depth-resolved attenuation mapping of the structural B-scan helps better visualization of the borders between the epithelium and the lamina propria.

The purpose of the current study was to assess connective tissue changes in the vaginal wall under prolapse and after treatment with a neodymium (Nd:YAG) laser, using CP OCT with depth-resolved attenuation mapping. To the best of our knowledge, this study is the first to demonstrate B-scan attenuation maps of human vaginal wall tissue in various conditions in the co- and cross-polarization channels. In future, this technique might also be useful for in vivo detection of early stages of vaginal wall prolapse and assessment of the efficacy of laser treatments.

## 2. Materials and Methods

### 2.1. Patients and Samples

The study involved three groups of patients from whom 26 freshly excised samples of the vaginal wall were taken for ex vivo CP OCT and histological examination. The patients’ ages ranged from 35 to 50 years old. The first group was the age norm (*n* = 8), where surgical intervention in the pelvic area was performed for reasons other than prolapse. The second group was stages I–II vaginal wall prolapse (anterior and/or posterior localization) without preoperative laser treatment (*n* = 8). The stage of prolapse was estimated according to POP-Q (Pelvic Organ Prolapse Quantification system) [45]). The third group was stages I–II vaginal wall prolapse with preoperative laser treatment (*n* = 10). Patients with prolapse of the vaginal wall underwent colporrhaphy to correct the POP. All samples were obtained by incisional biopsy of the vaginal wall in the region of the posterior fornix.

This study was approved by the institutional review board of the Privolzhsky Research Medical University (Protocol #13 from 7 July 2021, protocol #2 from 4 February 2022). This trial was prospectively registered on Clinical trial. gov, NCT05000957, registered 11 August 2021, https://clinicaltrials.gov (accessed on 24 September 2023). All the patients included in the study provided written informed consent.

### 2.2. Intravaginal Nd:YAG Laser Treatment Protocol

In this study, Nd:YAG laser treatment was used in addition to surgical interventions. The laser treatment of the vaginal wall was performed by “Magic Gyno” laser—a modification of “Magic Max” laser (“MeLSyTech”, Ltd., Nizhny Novgorod, Russia). The laser’s technical characteristics were as follows: Nd:YAG laser type; wavelength 1064 nm; Q-switch mode; the energy of one pulse up to 1.5 mJ; the duration of one pulse 20–200 ns; the pause between pulses 30 μs.

The laser treatment included two steps: the first one involved treatment of all vaginal walls in a circle with a conical mirror handpiece, and the second one involved targeted treatment of specific vaginal walls (anterior, posterior or lateral, depending on the clinical situation) with a corner mirror handpiece. The laser treatment procedure is described in more detail in [30]. Three laser procedures were performed with an interval of 4–6 weeks. Surgery with biopsy was performed 1–2 months after the last treatment when the effect of stimulating collagen synthesis was achieved.

### 2.3. OCT System

A spectral-domain OCT system (BioMedTech LLC, Nizhny Novgorod, Russia) that provides two image acquisition in co- and cross-polarizations was used for vaginal wall visualization. This OCT system (product license No FCP 2012/13479 of 30 May 2012) has a common-path interferometric layout that operates at a 1310 nm central wavelength. Axial resolution is 10 µm, lateral resolution is 15 μm, scanning depth—2 mm in air, and scanning speed—20,000 A-scans per second. The system performs 2D lateral scanning with a range of 2.4 × 2.4 mm^2^ to obtain the 3D distribution of backscattered light. Scanning is performed in contact mode and takes 26 sec. The system combines traditional structural OCT and polarization modes. As a result, CP OCT images in two virtual channels are recorded simultaneously: an image in co-polarization (reflected light with a polarization state parallel to the initial polarization state) and an image in cross-polarization (reflected light with changed polarization which is orthogonal to the initial one) channels [46]. CP OCT is aimed at obtaining the information contained in the cross-polarization channel, which allows one to visualize the birefringence of the tissue from optically anisotropic structures, as well as coherent cross-polarization backscattering on non-spherical particles and particles with dimensions much larger than the wavelength. In the connective tissue of the vaginal wall, such structure is represented by elongated collagen fibers and bundles.

**Figure 1:**
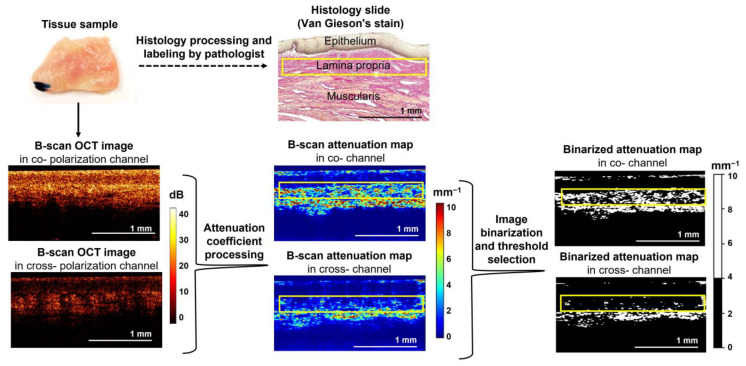
Overview of the methodology for construction and processing of the dataset. Each sample was scanned with the CP OCT system, and B-scans were obtained. After that, attenuation coefficient maps were built and compared to the labeled cross-sectional histology to define and analyze the ROI within the lamina propria (yellow rectangles). Two parameters—median values of the attenuation coefficient and percentage ratio with values above/below a certain threshold within the same ROI—were calculated. A threshold equal to 4 mm^−1^ was proposed to binary-separate pixels with high values from low ones (see binarized attenuation maps).

As was previously shown [40], the OCT signal in two polarization channels can differ depending on the quantity and density of collagen content. In the normal vaginal wall, the OCT signal from lamina propria in both polarization channels is of the same strength due to the dense arrangement of collagen fibers/bundles (log-scale OCT image is not presented). In the case of the I–II stage prolapse, there could be a noticeable difference in signal strength between the two channels in the original log-scale B-scan OCT image (Figure 1). This difference is attributed to the presence of a reduced number of collagen fibers/bundles in lamina propria and their looser arrangement. The transition from the standard log-scale images to the attenuation coefficient distribution allows highlighting of this difference, which may arise from the nature of the OCT signal, as discussed below.

From each sample, 4–8 CP OCT images were obtained. The number of images depended on the sample size. Scanning was carried out with image overlay to obtain data from the entire surface under study. In total, over 200 CP OCT images of vaginal wall tissue were obtained. The imaging results were confirmed by histopathology.

### 2.4. CP OCT Data Processing and Quantitative Analysis

The quantitative processing of the 3D CP OCT images was based on calculating the attenuation coefficients in two polarization channels: in the co-channel, Att(co), and in the cross-channel, Att(cross). Calculation of the attenuation coefficient (µ) is an objective and generally accepted approach that describes the loss of OCT signal with depth due to scattering and absorption of probe radiation and is used in the evaluation of a wide variety of tissues [47]. This metric allows improved visualization of biological structure and an objective differentiation of normal and pathological tissues.

For attenuation coefficient calculation, we used the depth-resolved approach proposed by Vermeer in [48] and modified to account for the noise with the non-zero mean present in the distributions of the measured absolute values of the OCT images and allowed avoidance of systemic attenuation coefficient estimation bias [49,50]. The non-zero additive noise in the images’ background arises from the usage of the absolute value of the OCT image for processing, which leads to the non-zero mean additive noise. To reduce the effect of speckle noise on the local attenuation coefficient estimation, the volume of the measured OCT data was averaged in a local 3 × 3 × 3-pixel window before the attenuation coefficient estimations. While the physical model underlying the calculations does not account for the polarization effects, we applied the equations for the attenuation coefficient estimation for the signal in the cross-channel as well, as we expected that Att(cross) may provide us with more information about the connective tissue changes in the vaginal wall under prolapse and after Nd:YAG laser treatment.

As shown in [48], the attenuation coefficient could be estimated as:

(1) $$\mu_{i}^{att}=\frac{I_{i}}{2\Delta \sum_{i+1}^{i_{\max}}I_{i}}$$

where *I_i_* is the noise-free OCT signal amplitude in the co- or cross-channel, *µ_i_^att^* is the corresponding specimen attenuation coefficient estimation, *i*—axial measurement number, *i*_max_—total number of pixels in axial direction, and Δ is the pixel size along the axial dimension.

Due to the presence of the no-zero additive noise in the experimental OCT signal amplitude distribution, the direct application of Equation (1) to the measured OCT signal will lead to the following estimation:

(2) $$\mu_{i}^{est}=\frac{I_{i}+N_{i}}{2\Delta \sum_{i+1}^{i_{\max}}I_{i}+N_{i}}$$

where *N_i_* is the additive noise, and *µ_i_^est^* is the attenuation coefficient estimation obtained from the OCT data with the presence of noise. To mitigate the effect of the noise, in [49], it was proposed to reorganize Equation (2) as follows to improve the estimation:

(3) $$\begin{array}{l} \mu_{i}^{est}=H_{i}\cdot \mu_{i}^{att}+N_{i}^{\mu },\\ where\\ H_{i}=1-\frac{\sum_{i+1}^{\infty }N_{j}}{\sum_{i+1}^{\infty }I_{j}+\sum_{i+1}^{i_{\max}}N_{j}}=1-\frac{\langle N\rangle \cdot (i_{\max}-i)}{\sum_{i+1}^{\infty }I_{j}+\sum_{i+1}^{i_{\max}}N_{j}}\\ N_{i}^{\mu }=\frac{N_{j}}{2\Delta \sum_{i+1}^{i_{\max}}\left(I_{j}+N_{j}\right)}=\frac{\langle N\rangle }{2\Delta \sum_{i+1}^{i_{\max}}\left(I_{j}+N_{j}\right)} \end{array}$$

where ⟨*N*⟩ is the mean noise amplitude.

Such reorganization of the equation allows one to obtain the estimation of the attenuation coefficient according to Equation (1) from the estimation according to Equation (2):

(4) $$\mu_{i}^{att}=\frac{H_{i}\cdot SNR_{i}^{\mu }}{{\left|H_{i}\right|}^{2}\cdot SNR_{i}^{\mu }+1}\cdot \mu_{i}^{est}$$

where $SNR^{\mu }{}_{i}$ is the local signal-to-noise ratio (*SNR*).

In order to provide an accurate comparison with histological results and to be able to analyze the lamina propria layer, distributions of the attenuation coefficients within the depth were plotted as cross-sectional (B-scan), color-coded maps for both polarization channels (Figure 1, middle column). The color set of each map reflects the values of the corresponding attenuation coefficient: areas with a high signal decay rate are represented by shades of yellow and red, while areas with low attenuation use shades of blue and light blue. This color-coded map representation (Figure 1, middle column) enhances the optical contrast of the original log-scale OCT images (Figure 1, left column), such as the boundaries of the tissue layers, the depth of disappearance of the useful signal and the distinction between low and high signal areas, and thus facilitates interpretation and quantification of the data. Therefore, subsequent calculations could be carried out using only the attenuation coefficient maps.

The quantitative processing consisted of the calculation of two parameters for each polarization channel: (1) the median values of the attenuation coefficients, which were automatically calculated from among all the values in the selected region of interest (ROI) and (2) the percentages with values above/below a certain threshold (4 mm^−1^) within the same ROI. For this, in each of the created color-coded attenuation maps, a rectangular ROI was manually selected, first in the cross-channel, and then a region of the same dimensions was used in the co-channel. Each ROI was located in the lamina propria layer to enable exact comparison with the histological data (see Figure 1, yellow rectangles). Lamina propria layer thickness is at least 250–300 µm; therefore, this layer is well distinguished in OCT images in both channels. The ROIs were typically ~300 × 2000 μm in size. Generally, for each 3D CP OCT image, three cross-section attenuation maps were quantitatively assessed to better represent the consistency of the optical properties of the sample, considering its morphological heterogeneity and the locality of some pathological processes.

To calculate the second parameter, a threshold value of the OCT signal attenuation coefficient of 4 mm^−1^ was proposed, which was selected empirically in such a way as to divide all pixels on the attenuation maps in the ROI into two groups: (1) coefficient values ≥4 mm^−1^, which were considered “high” and corresponded to an area with a dense arrangement of collagen bundles; and (2) coefficient values < 4 mm^−1^, which were considered “low” or noisy and were consistent with loose arrangements of the collagen bundles, or were regions containing lymphatic vessels. Thus, this parameter, based on the ratio of high and low coefficient values, allowed us to separate areas of dense arrangements of collagen bundles from areas with loose arrangements or sites of lymphatic vessels.

### 2.5. Histological Examination

After CP OCT imaging of the vaginal wall samples, the scanning area was marked with histological ink for correlation with the histological plane. Histological sections were prepared according to the standard procedure and stained with hematoxylin and eosin for assessment of tissue structure. Van Gieson’s stain was used for assessment of the collagen bundles (takes on bright red color) to observe their changes during the development of prolapse and after laser exposure in the subepithelial layer or lamina propria. On histological preparations stained with Van Gieson’s, three states of collagen bundles were assessed: thickness (thick and thin), density and orientation of their location. The values and distribution of the attenuation coefficient values in the cross-polarization channel depended on these three states of collagen bundles. Since the lamina propria is known to contain lymphatic vessels [51], we additionally used immunohistochemical examination to visualize them. An immunohistochemically examination was performed with a monoclonal antibody to a marker of lymphatic endothelial cells Podoplanine (ab236529; Abcam, Cambridge, UK) in order to identify lymphatic vessels. Histological sections were examined using the EVOS M7000 Imaging System (Thermo Fisher Scientific Inc., Waltham, MA, USA) in transmitted light.

### 2.6. Statistics

The variables for statistical intergroup comparison were the Att(co) and Att(cross) coefficients calculated from CP OCT images. The results are expressed as Me [Q1; Q3], where Me—is the median of the analyzed parameter and [Q1; Q3] are the 25th and 75th percentile values, respectively. The Mann–Whitney U-test with Bonferroni correction was used to evaluate the statistical significance, with a *p*-value of less than 0.05 considered statistically significant. The box plots and calculations were carried out using the Prism 8.0.2 statistical software (GraphPad Software, San Diego, CA, USA).

## 3. Results

### 3.1. Correspondence of Depth-Resolved Attenuation Maps with the Histology for Vaginal Wall at Different States

**Figure 2:**
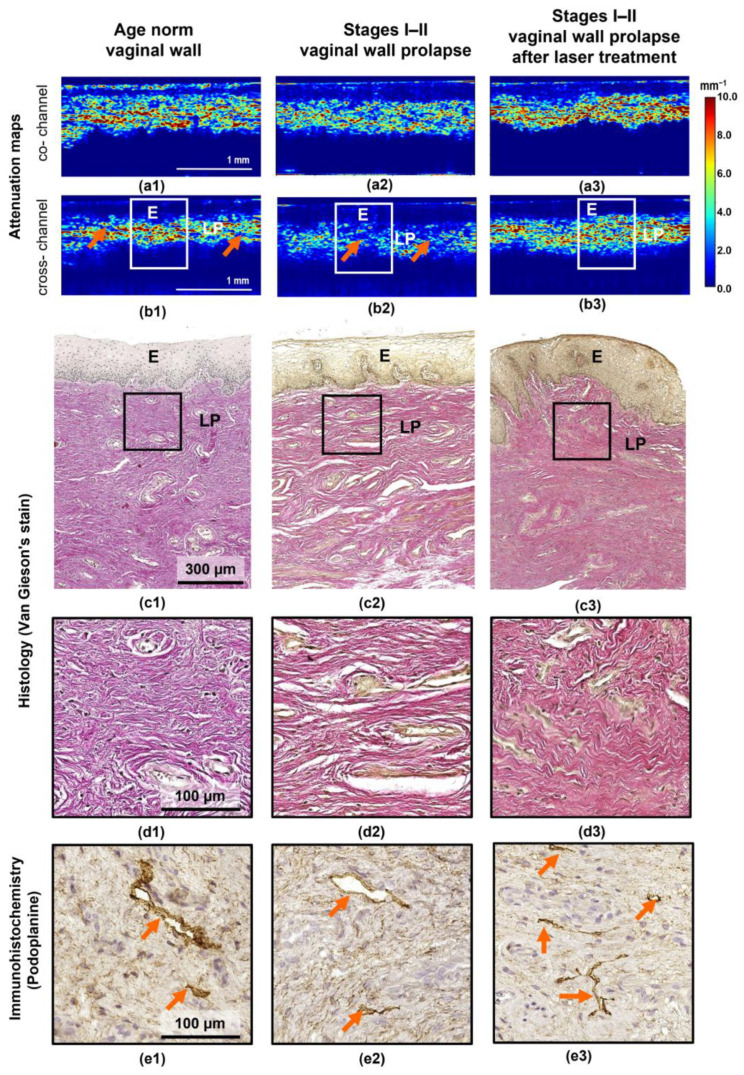
Attenuation coefficient mapping for the vaginal wall at different states: age norm (left column, (**a1**–**e1**)), stages I–II prolapse (central column, (**a2**–**e2**)), stages I–II prolapse after laser treatment (right column, (**a3**–**e3**)). Depth-resolved attenuation maps in co- (**a1**–**a3**) and cross- (**b1**–**b3**) channels. (**c1**–**c3**) Overview of histological images stained with Van-Gieson’s show two-layer tissue architecture with a sharp border between the glycogenated epithelium and the lamina propria underlying it (×200); (**d1**–**d3**) an enlarged area of the subepithelial region of the lamina propria showing different thicknesses and patterns of arrangement of collagen bundles (×1000); (**e1**–**e3**) immunohistochemical examination with Podoplanin; the area of the lamina propria is demonstrated, where orange arrows indicate lymphatic vessels (×1000). In the attenuation coefficient maps, the white rectangles are the areas corresponding to the histological images in (**c1**–**c4**), and the orange arrows indicate lymphatic vessels. Abbreviations: E—epithelium, LP—lamina propria.

In the first stage of this study, attenuation coefficient maps in the co- and cross-polarization channels and corresponding histological slices of the vaginal wall in different states were visually analyzed and described. Figure 2 presents typical images for the age norm (a1–e1, left column), stage I–II prolapse without treatment (a2–e2, central column) and corresponding prolapse after laser treatment (a3–e3, right column). It can be seen that all the images (Figure 2(a1–c3)) display two-layer architecture with a clear boundary between the epithelium and the lamina propria. Notably, the lamina propria has a different scattering and polarization ability depending on the state and distribution of the collagen bundles in the different pathogenic conditions of the vaginal wall.

In the case of the age norm, the attenuation coefficient maps in the co- and cross-channels (Figure 2(a1,b1)) show a clear boundary between the epithelium with low attenuation coefficients and the relatively high attenuation coefficients in the lamina propria region. The high values of attenuation coefficient in the cross-channel of the lamina propria are due to the dense arrangement of thick and thin collagen bundles without any preferential orientation; the spaces between the bundles are very small (Figure 2(d1)). The heterogeneous distribution of values is due to lymphatic vessels (orange arrows in Figure 2(e1)), which do not scatter the signal and are represented on the attenuation coefficient maps as slit-like structures with low values, penetrating the connective tissue (orange arrows in Figure 2(b1)).

In stages I–II of vaginal wall prolapse, the attenuation coefficient maps show relatively lower attenuation coefficients in the cross-channel (Figure 2(b2)) in the area of connective tissue when compared with the age norm (Figure 2(b1)). This is consistent with the histological images, where the lamina propria is characterized by loosely arranged, thin collagen bundles that have a predominant direction aligned along the epithelium; the spaces between the collagen bundles are expanded (Figure 2(c2,d2)). While no changes were observed in the number of lymphatic vessels, their lumens were expanded (Figure 2(e2)) compared to the age norm (Figure 2(e1)). Meanwhile, the attenuation map in the co-channel (Figure 2(a2)) does not reflect the true state of the vaginal wall connective tissue during prolapse and therefore does not help in distinguishing it from the age norm. In some of the studied vaginal wall prolapse samples, those with a very thin lamina propria, the attenuation coefficient maps clearly visualized the deeper muscle layer, which had a higher attenuation coefficient compared to the lamina propria (see example in Figure 1, right column).

Nd:YAG laser treatment leads to changes in both the epithelium and the lamina propria (Figure 2(a3–e3)). In the attenuation coefficient maps (Figure 2(a3,b3)), the border between the epithelium and the lamina propria can be seen to have become uneven. Histologically, this corresponds to the epithelial ridges that protrude into the lamina propria (Figure 2(c3)). A significant increase in the attenuation coefficient values is therefore observed in the lamina propria (Figure 2(b3)) compared with its state without treatment (Figure 2(b2)). These changes are better visualized in the attenuation coefficient map in the cross-channel (Figure 2(b3)) than in the co-channel (Figure 2(a3)). It is important to note that a deeper OCT signal was visualized in the cross-channel than in the co-channel. Histologically, this corresponds to the formation of a dense network of thickened collagen bundles with practically no space between them, indicating an increase in the uniformity of their alignment during the remodeling of the connective tissue in response to the laser treatment (Figure 2(d3)). In addition, the formation of dense connective tissues could lead to a predominance of lymphatic vessels with collapsed lumens (Figure 2(e3)), which also explains the homogeneity of the attenuation coefficient maps.

### 3.2. Quantitative Analysis of Attenuation Maps under the Studied Conditions of the Vaginal Wall

**Figure 3:**
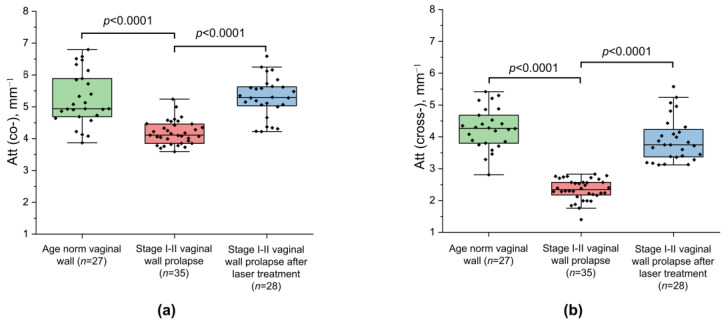
Boxplots for (**a**) Att(co) and (**b**) Att(cross) coefficients counted for three states of vaginal wall tissue. Center line in the boxes—median; box limits—25th and 75th percentiles; whiskers—minimum and maximum values within the 1.5× interquartile range of the first and third quartile. Segment indicates a statistically significant difference between the study groups (Mann–Whitney U-test with Bonferroni correction for multiple comparisons), where *p*—the magnitude of the statistical significance of the differences between states of vaginal wall tissue, and *n*—is the number of examined images for each group.

Figure 3 presents diagrams of the median values of the Att(co) and Att(cross) coefficients calculated for the ROIs within the lamina propria for three groups of patients: age norm (number of images *n* = 27), stage I–II prolapse (*n* = 35) and stage I–II prolapse post laser treatment (*n* = 28).

The stages I–II vaginal wall prolapse are characterized by a statistically significant decrease in the median values of both the Att(co) and Att(cross) coefficients compared to the age norm (4.1 [3.9; 4.4] mm^−1^ vs. 4.9 [4.7; 5.8] mm^−1^ and 2.3 [2.1; 2.5] mm^−1^ vs. 4.2 [3.8; 4.6] mm^−1^, respectively; *p* < 0.0001) (Figure 3a,b; green and red boxes). At the same time, the vaginal wall prolapse is characterized by significantly lower values of Att(cross) coefficients compared with the Att(co) coefficients. As shown above, this is due to the fact that in prolapse cases, the connective tissue is characterized by loosely arranged, thin collagen bundles, leading to a lower signal attenuation rate with depth in the cross-channel (see Figure 2). In addition to these collagen changes, the presence of a large number of lymphatic vessels in the connective tissue can also lead to a decrease in the mean ROI values of both the Att(co) and Att(cross) coefficients (see Figure 2(a2,b2)).

After Nd:YAG laser treatment, the values of both the Att(co) and Att(cross) coefficients considerably increased and constituted 5.3 [5.0; 5.6] mm^−1^ and 3.8 [3.4; 4.2] mm^−1^, respectively (Figure 3a,b; blue boxes). Here, vaginal wall prolapse after laser treatment statistically differs from vaginal wall prolapse without laser treatment, accounting for both the Att(co) and Att(cross) coefficients (*p* <0.0001) (Figure 3b). At the same time, calculating the Att(cross) coefficient allows us to more reliably identify areas with an increased density of collagen bundles (Figure 3b; blue box).

Overall, Figure 3a indicates that the variability of the median values for the Att(co) coefficient for individual samples may be even stronger than the difference between groups, even if the inter-group difference is statistically significant (*p* < 0.0001). The Att(cross) coefficient in Figure 3b demonstrates a lower variability of values and, accordingly, makes it possible to more reliably differentiate all conditions of the vaginal wall tissue studied here.

**Figure 4:**
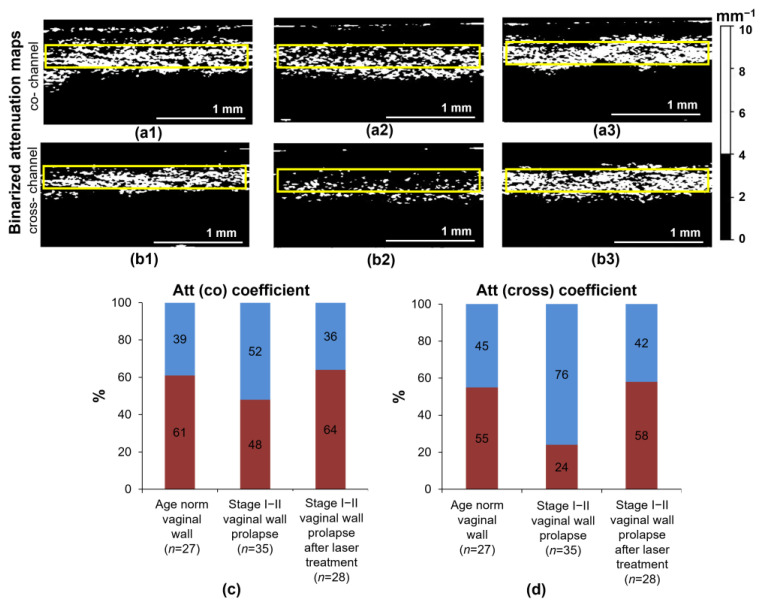
Binarized attenuation maps in co- (**a1**–**a3**) and cross- (**b1**–**b3**) channels for three states of the vaginal wall: age norm (**a1**,**b1**), stage I–II prolapse (**a2**,**b2**) and stage I–II prolapse after Nd:YAG laser treatment (**a3**,**b3**) and its quantitative assessment (**c**,**d**) by calculation of the percentage of pixels with high (red color) and low (blue color) Att(co) (**c**) and Att(cross) (**d**) values (*n*—the number of examined images for each group). Binarization was performed using a threshold of 4 mm^−1^.

The results of calculating the parameter, percentages of pixels with low (<4 mm^−1^) and high (≥4 mm^−1^) attenuation values, are presented in Figure 4. This parameter allows tracking of the change in the number of thick, densely packed collagen bundles, excluding areas occupied by lymphatic vessels or thin, multidirectional collagen bundles. Figure 4a,b show examples of binarized attenuation coefficient maps with the applied threshold (4 mm^−1^) for three states of the vaginal wall: age norm (a1–b1), stage I–II prolapse (a2–b2) and stage I–II prolapse after Nd:YAG laser treatment (a3–b3). The results demonstrate that changes in collagen state in stage I–II prolapse and after Nd:YAG laser treatment are most noticeable in the cross-channel (Figure 4(b2,b3)). Quantitatively, the percentage of the high Att(cross) values in stage I–II prolapse without laser treatment is ~2 times lower compared to the age norm group (24% vs. 55%, respectively) (Figure 4d). In the co-channel, there are minor changes between these two states: 48% in stage I–II prolapse without laser treatment vs. 61% in the age norm (Figure 4c). Further, after Nd:YAG laser treatment, the percentage of the high values of both the attenuation coefficients tend to approach those at the age norm, being 58% in the cross-channel and 64% in the co-channel (Figure 4c,d). The content of the scattering component for the laminae propriae of stage I–II prolapse after treatment does not differ greatly from the age norm in the percentages of high and low values for both the Att(co) (64% and 61%, respectively) (Figure 4c) and the Att(cross) (58% and 55%, respectively) coefficients (Figure 4d), which indicates that the state of the collagen framework in the connective tissue after laser treatment is close to normal.

Thus, this once again demonstrates that for the studied conditions of the vaginal wall, the difference in the values of the analyzed parameters is more significant in the cross-channel than in the co-channel.

## 4. Discussion

In this paper, for the first time, we calculated the attenuation coefficient from CP OCT images of human vaginal wall tissue in different states: age norm, stage I–II prolapse and stage I–II prolapse after Nd:YAG laser treatment. Attenuation coefficient calculation from OCT data allowed us to achieve improved biological structure visualization and objectively differentiate between tissues of different morphology [41,42,43,44]. In the present study, the depth-resolved method for attenuation coefficient was used for the first time to evaluate connective tissue changes in the vaginal wall. As has been shown in [49], the depth-resolved method for attenuation coefficient calculations can identify additional structural features. Unlike more commonly used methods based on the linear fit of the logarithm of the signal, the method utilized in the study avoids axial resolution deterioration from the selection of the fitting range, decreases the variance of the attenuation coefficient estimation of the optically uniform specimen and avoids biases from the poor choice of fitting range in the case of the layered object. For the first time, cross-sectional (B-scan) color-coded maps to represent attenuation coefficient value distributions have been built for two polarization channels for the vaginal wall. The construction of B-scan attenuation maps is most applicable for the visualization of layered biological tissues, including the vaginal wall. In our study, this allowed us to clearly distinguish the epithelium from the underlying lamina propria and assess changes in collagen bundles in the latter layer. Previously, attenuation mapping has predominantly been used in the en face plane, which is preferable for non-layered structures, such as, for example, tumors or inflammations [41,43].

In our previous paper [30] describing quantification of changes in the state of collagen bundles localized in the laminae propria of the same studied groups, C-OCE methods and quantitative morphological analysis of the collagen bundles (local thickness, uniformity and orientation) were used. In the case of vaginal wall prolapse, we observed a statistically significant decrease in tissue stiffness and its increase after Nd:YAG laser treatment. The results of quantitative morphological analysis also showed a decrease in the local thickness of collagen bundles and the uniformity of their arrangement during stage I–II prolapse. However, after Nd:YAG laser treatment, an increase in the local thickness of the collagen bundles, a change in their orientation and an increase in the uniformity of their arrangement were demonstrated. In the current study, we did not carry out morphometry; however, we visually observed similar processes in the connective tissue: a reduced density and thinning of collagen bundles during prolapse, an increase in the thickness of the collagen bundles and an increase in their density after laser treatment. It should be noted that CP OCT, in comparison with C-OCE, has greater potential to be used for further, more rapid derivation of 2D or 3D images of the vaginal wall tissue in vivo with higher resolution (~10–15 µm) without additional compression of the tissue and using a reference layer. In addition, compared to our previous C-OCE study [30], the number of study samples was increased, which confirmed our results on the nature of the changes in the collagen bundles in vaginal wall prolapse without and after laser treatment. Our study is also consistent with studies using electron microscopy, in which it was observed that collagen fibrils lost their normal parallel structures in the affected vaginal tissue and also had large gaps between them [52,53].

In this study, we have demonstrated, for the first time, specific changes in the collagen bundles of the vaginal wall connective tissue are associated with the decrease in its scattering and polarization properties. The level and nature of cross-polarization backscattering in the cross-channel on OCT images and, accordingly, the attenuation coefficient of the OCT signal in the cross-channel mainly depended on the number, orientation and density of collagen bundles in the lamina propria of the vaginal wall. This is an important factor in the development of the early stages of vaginal wall prolapse and in assessing the restoration of the mechanical properties of the vaginal wall after laser treatment. Using CP OCT, it is possible to acquire a clear distinction between areas with loosely arranged collagen bundles and densely arranged ones based on the ability of collagen bundles to cross-polarization backscatter. Two parameters within the lamina propria were targeted and calculated: the median value and the percentages of high (>4 mm^−1^) and low (<4 mm^−1^) attenuation coefficient values. In this case, all tissue structures that do not generate an OCT signal (mainly lymphatic vessels or spaces between collagen bundles) or generate a weak signal (thin collagen bundles) fell into the category of structures with an attenuation coefficient value of <4 mm^−1^. The median values of the Att(co) and Att(cross) coefficients in the lamina propria were significantly (*p* < 0.0001) lower in the stage I–II prolapse compared with those in the age norm (Figure 3). This was associated with thinner collagen bundles and large slit-like spaces between them in the case of prolapse of the vaginal wall. After the laser treatment, significantly higher median values of the Att(co) and Att(cross) coefficients were noted, compared to the vaginal wall prolapse without treatment (*p* < 0.0001). This was associated with an increase in the local thickness of the collagen bundles, changes in their orientation and an increase in the uniformity of their arrangement, leading to more noticeable polarization effects. The difference in the spatial localization of the high and low regions in the attenuation maps in the cross-channel for the age norm and the stage I–II prolapse, both without and after laser treatment, can additionally be useful for differentiation between these three conditions. It was shown that when calculating the percentage of high (≥4 mm^−1^) and low (<4 mm^−1^) attenuation coefficient values (Figure 4), the Att(cross) coefficient better reflects the presence of the densely arranged and thickened collagen bundles and distinguishes them from areas occupied by loosely arranged collagen bundles or dilated lymphatic vessels. Significantly lower values were found for stage I–II prolapse compared to the age norm (24% vs. 55%), with a return to normal values after Nd:YAG laser treatment (58%). This new knowledge—the significant difference between stage I–II prolapse and the age norm and stage I–II prolapse after Nd:YAG laser treatment—holds promise for using CP OCT both to diagnose early stages of vaginal wall prolapse and to enable quantitative monitoring of the changes in the lamina propria of the vaginal wall after Nd:YAG laser treatment.

These results are consistent with the results of earlier studies involving calculations of the attenuation coefficients of OCT signal in the tissues of the pelvic organs. In particular, a decrease in the attenuation coefficient for cancerous ovaries when compared with normal or benign ovaries has been shown [44] that could have similarly originated from the remodeling of collagen fibers during the progression of such ovarian cancers [54]. Using CP OCT technology, the quantitative parameters of the attenuation coefficients for dermal lesions in vulvar lichen sclerosus have also been established, which makes it possible to detect the disease in the case of an early lesion based on a decrease in the values of the attenuation coefficients due to decreases in the thickness of collagen bundles as well as the presence of dermal edema [42]. Previously, our group has also demonstrated the use of CP OCT to visualize the contours of lymphatic vessels more clearly using depth-resolved attenuation mapping in vulvar lichen sclerosus [49]. In this study, while clear contours of the lymphatic vessels were also well visualized in the attenuation maps of the vaginal wall, we found no significant difference in the percentage areas involved between stage I–II prolapse and the age norm and stage I–II prolapse after Nd:YAG laser treatment.

In this paper, it is also worth noting the positive dynamics of changes in the condition of the vaginal mucosa after neodymium non-ablative laser treatment, which may, in the near future, compete with treatments traditionally used in the clinic today, as well as with microablative laser therapy approaches [17,18,19] for treating gynecological and urological problems. At the same time, CP OCT with depth-resolved attenuation mapping may provide an objective method for monitoring the efficiency of Nd:YAG laser treatment of the early stages of vaginal wall prolapse since the depth of the OCT imaging (~2 mm) corresponds to the depth of the laser exposure. It has been shown that the nature of the remodeling of the vaginal wall tissues after Nd:YAG laser exposure can be determined by an increase in the quantitative parameters of the attenuation coefficients in the area of the lamina propria, thus indicating an intensification of the regenerative reactions of the tissue. Certainly, further accumulation of CP OCT data and rigorous statistical analysis are required to verify the diagnostic value of the encouraging results presented above, based on our examination of a set of 26 samples comprising three conditions of the vaginal wall. However, even these data demonstrate that, in addition to the conventionally discussed mean attenuation values, analysis of the CP OCT-based spatial localization of high and low values of the attenuation coefficient regions opens very promising prospects for distinguishing between effective and ineffective laser treatment.

In the future, we anticipate that the CP OCT can be a robust method for detecting early stages of vaginal wall prolapse in vivo based on reducing the attenuation coefficient values in the cross-channel. In addition, the use of CP OCT will allow us to conduct dynamic monitoring of the effectiveness of Nd:YAG laser treatment of prolapse and to identify patients who can undergo repeated laser therapy courses to prevent a relapse of POP. However, it should be taken into account that during CP OCT examination of the vaginal wall in vivo, the values of attenuation coefficients may differ slightly (be lower) from the values obtained during ex vivo examination. We believe that the main reason for this difference is the fullness of blood and lymphatic vessels in living tissue. The presence of large dilated lymphatic vessels in the lamina propria, which have a signal at the noise level when calculating the attenuation coefficient within this layer, leads to a decrease in its average value in in vivo calculations compared to ex vivo tissue, where the contribution of low values from collapsed lymphatic vessels to the overall signal is not that significant. As for blood vessels, in vivo, they are characterized by a high level of co-channel backscatter, comparable to surrounding tissue (therefore, they are indistinguishable from other structures in structural OCT images), and the absence of cross-channel scattering, which reduces the average attenuation coefficient values in the cross-channel. In ex vivo tissue imaging, blood vessels have narrowed lumens, which virtually excludes these areas from the calculations and leads to an increase in the attenuation coefficient in the cross-channel, with almost no effect on the signal in the co-channel. Unlike vessels, collagen fibers do not significantly change their scattering properties before or after tissue excision. Therefore, we demonstrate the ability of the CP OCT method to assess the number and organization of collagen bundles in this pathology and the ability to monitor changes in their number and location after laser treatment. In the future, we plan to compare CP OCT data of the vaginal wall in in vivo and ex vivo studies.

One limitation of this study is that some of the early stage prolapse of the vaginal wall are focal; thus, the selected OCT B-scans in these cases may represent a mixture of the diseased and normal tissues. Moreover, given the likelihood of heterogeneous areas of connective tissue remodeling in the early stages after restoration of the anisotropic properties, at later dates, such differences will probably not be so noticeable. Therefore, in the early stages of monitoring the effectiveness of treatment in the future, it will be more appropriate to use an intravaginal OCT probe [36], which could enable examination of several different sections of the vaginal wall tissue and accordingly exclude the need for invasive (punch or excision biopsy) manipulations in such patients. Current efforts are underway for the construction and implementation of a circumferential-scanning intravaginal CP OCT probe for in vivo imaging of the vaginal wall.

In summary, the present study provides a new diagnostic approach both to non-invasive early detection of prolapse and to monitoring the efficiency of Nd:YAG laser treatment based on the assessment of the attenuation coefficients calculated from real-time CP OCT data. The results obtained in this study have shown several advantages of using attenuation coefficient maps in the cross-channel over conventional log-scale OCT images and attenuation coefficient maps in the co-channel. Firstly, they better reflect the true state of the lamina propria connective tissue. Secondly, the contours of the abundant low-attenuating slit-like structures (lymphatic vessels) located in the lamina propria are more clearly represented. Thirdly, the values of the Att(cross) coefficient in the tissues can themselves be used to provide an objective diagnostic parameter for the condition of the connective tissue.

## 5. Conclusions

In this study, we have shown that the use of optical attenuation coefficients of the OCT signal in two polarization channels and the construction of color-coded attenuation maps in the B-scan projection provide both an effective way to present CP OCT images of the vaginal wall tissue and an objective method for quantitative assessment of the OCT signal. It has also been shown that with depth-resolved attenuation mapping, there are large statistical differences (*p* < 0.0001) that can be used to distinguish OCT images of vaginal wall prolapse from age norm and from prolapse after Nd:YAG laser treatment, based on calculation of the median values of the attenuation coefficient in both channels. At the same time, attenuation maps of vaginal wall prolapse in the cross-channel better reflect the true state of the connective tissue of the lamina propria compared to conventional OCT images and the attenuation coefficient in the co-channel. The calculated percentage of high (≥4 mm^−1^) and low (<4 mm^−1^) attenuation coefficient values in the cross-channel more reliably reflect the state of the connective tissue by distinguishing between areas of loose and densely arranged collagen bundles. Therefore, the obtained results highlight the great potential of the CP OCT method with depth-resolved attenuation mapping for in vivo assessment of the state of the lamina propria connective tissue of the vaginal wall both under prolapse and after Nd:YAG laser treatment.

## Data Availability

The data are not publicly available due to proprietary rules.
